# Genome-wide identification of cold responsive transcription factors in *Brassica napus* L

**DOI:** 10.1186/s12870-020-2253-5

**Published:** 2020-02-06

**Authors:** Liping Ke, Weixia Lei, Weiguang Yang, Jinyu Wang, Janfang Gao, Jinhua Cheng, Yuqiang Sun, Zhixiong Fan, Dongliang Yu

**Affiliations:** 1 Plant Genomics & Molecular Improvement of Colored Fiber Lab, College of Life Sciences and Medicine, Zhejiang Sci-Tech University, Hangzhou, 310018 China; 2 Crop Institute, Anhui Academy of Agricultural Sciences, Hefei, 230031 China; 3 Wenzhou - Kean University, Wenzhou, 325060 China

**Keywords:** Cold stress, Transcription factor, *Brassica napus*, Short day

## Abstract

**Background:**

Cold stress is one of the primary environmental factors that affect plant growth and productivity, especially for crops like *Brassica napus* that live through cold seasons. Till recently, although a number of genes and pathways involved in *B. napus* cold response have been revealed by independent studies, a genome-wide identification of the key regulators and the regulatory networks is still lack. In this study, we investigated the transcriptomes of cold stressed semi-winter and winter type rapeseeds in short day condition, mainly with the purpose to systematically identify the functional conserved transcription factors (TFs) in cold response of *B. napus*.

**Results:**

Global modulation of gene expression was observed in both the semi-winter type line (158A) and the winter type line (SGDH284) rapeseeds, in response to a seven-day chilling stress in short-day condition. Function analysis of differentially expressed genes (DEGs) revealed enhanced stresses response mechanisms and inhibited photosynthesis in both lines, as well as a more extensive inhibition of some primary biological processes in the semi-winter type line. Over 400 TFs were differentially expressed in response to cold stress, including 56 of them showed high similarity to the known cold response TFs and were consistently regulated in 158A and SGDH284, as well as 25 TFs which targets were over-represented in the total DEGs. A further investigation based on their interactions indicated the critical roles of several TFs in cold response of *B. napus*.

**Conclusion:**

In summary, our results revealed the alteration of gene expression in cold stressed semi-winter and winter ecotype *B. napus* lines and provided a valuable collection of candidate key regulators involved in *B. napus* response to cold stress, which could expand our understanding of plant stress response and benefit the future improvement of the breed of rapeseeds.

## Background

Rapeseed (*Brassica napus* L.) is one of the most important economic crops in the world, with three main ecotypes, i.e., spring-, winter- and semi-winter types, are formed in the scenario of evolution and long-term cultivation to adapt to the diverse climates. Yangtze River basin is the most important ecological region for rapeseed production in China. Winter- and semi-winter rapeseeds are cultivated in Yangtze River basin with the seeds sowed from the end of September to mid-October, which means the growth and production of rapeseed are always threatened by the cold temperature. Therefore, determining the molecular mechanisms that rapeseeds have evolved to winter survival would not only make us understand more about the plant environmental adaptation but is also of great agricultural importance for China and other countries.

Cold acclimation, i.e., a period of exposure to low but non-freezing temperature, would increase the frost tolerance in a wide range of plants [1]. In this process, a series of plant biochemical and physiological features are adjusted and large-scaled alteration of gene expression are induced. Over the few past decades, great efforts have been made to unravel the molecular mechanisms involved in plant cold acclimation. CBFs/DREBs (cold-binding transcription factors/dehydration responsive element binding factors) -dependent signaling, the key and conserved regulatory mechanism of cold stress response, was characterized in many plants, with the components containing the CBFs and their activators (e.g., ICE1, CAMTA3 and BZR1/BES1) and repressors (e.g., MYB15, PIFs and EIN3) [2, 3]. Meanwhile, involvement of some CBF-independent regulatory pathways in cold response was also characterized in many plants, such as the plant hormones of auxin, abscisic acid, ethylene, gibberellins and jasmonic acid [4].

To date, many studies have investigated the physio-biochemical and molecular changes of cold stressed rapeseeds. In cell ultrastructure, pronounced modifications were observed in cold acclimation of *B. napus*, such as increased thickness of cell walls and invagination of plasma membranes [5]. Besides, extensive modulation in gene expression was also detected, including transcript accumulation for an arrange of genes, e.g., the cold-responsive (COR) genes *BN115* (COR15) and *BNCOR25* [6, 7], *BN59* (H^+^-ATPase subunit) [8], *hsp90* [9], multiple *CBF*s [10, 11] and a series of transcription factors (TFs) like APETALA2/ethylene response factors and NACs [12, 13]. More recent studies also indicated the potential roles of microRNAs and lncRNAs in rapeseed response to cold stress [14, 15]. However, there are still quite many remaining entangled, e.g., most of the previous researches were implemented in long-day photoperiod conditions, which were divorced from the short day condition in actual field production. Moreover, it remains unclear if the known mechanisms are conserved across accessions and in which manner those COR genes are regulated.

In the present work, we comparatively analyzed the transcriptomes of cold stressed semi-winter (158A) and winter type (SGDH284) rapeseeds after a seven-day cold treatment, with the purpose to reveal the conserved and key regulators in the cold response of rapeseed under short-day photoperiod.

## Results

### Identification and general features of differentially expressed genes (DEGs)

High-throughput sequencing generated an average of about 6.5 million qualified short reads from each samples of the semi-winter type (158A) and the winter type (SGDH284) *B. napus* lines, with around 70% of them were mapped to the reference genome (Additional file 1: Table S1). With the cutoff of FPKM ≥1 in at least one samples, expression of about 40,000 genes (~ 25% of the whole set) were detected in 158A (40,277) and SGDH284 (39,200). Notably, a greater number of expressed genes were detected in cold stressed 158A and SGDH284 than the controls, with the ratios of about 8.6 and 11.6%, respectively.

**Fig. 1:**
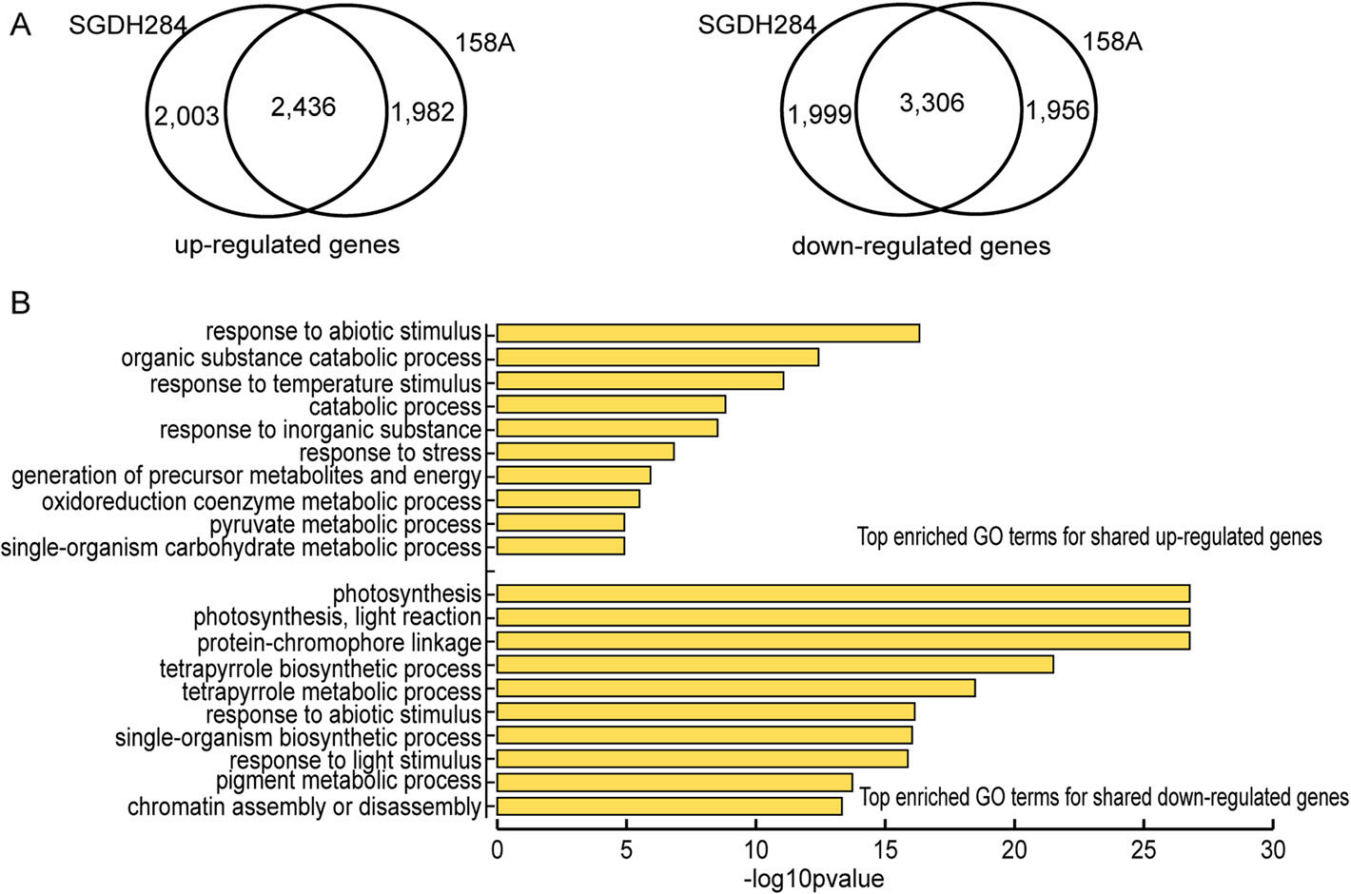
Differentially expressed genes in cold stressed *Brassica napus*. **a** Venn diagrams indicated the number of differentially expressed genes in 158A and/or SGDH284. **b** Function analysis of shared up- and down-regulated genes. Top 10 enriched GO terms were showed

After exposure to low temperature, about 10% of the whole set of genes (~ 10,000 genes) were differentially expressed in both 158A and SGDH284, including 2436 and 3306 genes that were consistently up- and down-regulated in both lines (Fig. 1a). Function enrichment analysis revealed that, the shared up-regulated genes were significantly enriched in mechanisms responsible for a wide range of environmental stresses and the catabolic processes related to energy production, while the shared down-regulated genes were enriched in biological processes associated with photosynthesis, stress response and chromatin organization (Fig. 1b). Functions of the line-specific DEGs were also analyzed, which revealed the remarkable differences in gene expression between cold stressed 158A and SGDH284. In the semi-winter line 158A, the down-regulated genes were significantly associated with the processes such as organonitrogen compound biosynthesis, microtubule-based movement, translation and DNA replication, whilst the up-regulated genes were mainly related to the stresses response and transporters. In contrast, down-regulated genes in the winter type line SGDH284 were mainly involved in stresses response and amino acid/sugar/sulfur compound metabolic processes, and the SGDH284 specific up-regulated genes were enriched in the processes related to the biosynthesis of constitute macromolecules and histone ubiquitination (Table 1). Further investigation revealed that significantly more genes involved in translation, DNA replication and regulation of cell cycle were identified in 158A specific down-regulated genes, as well as more genes associated with the ribosome biogenesis were found in the SGDH284 specific up-regulated gene set (Additional file 1: Table S2), suggesting the basic biological processes that maintained the cellular activities were more significantly inhibited in the semi-winter line 158A.

**Table 1:** Function enrichment analysis of line-specific differentially expressed genes in cold stressed semi-winter (158A) and winter type (SGDH284) *B. napus* plants

Class	Term_ID	Description	log10(*p*-value)
top10 enriched GO terms for line-specific down-regulated genes			
158A	GO:1901566	organonitrogen compound biosynthetic process	−12.42
	GO:1901564	organonitrogen compound metabolic process	−11.23
	GO:0007018	microtubule-based movement	−10.96
	GO:0006928	movement of cell or subcellular component	−10.82
	GO:0007017	microtubule-based process	−10.15
	GO:0006412	translation	−8.25
	GO:0043603	cellular amide metabolic process	−7.92
	GO:0044249	cellular biosynthetic process	−7.08
	GO:1901576	organic substance biosynthetic process	−6.82
	GO:0006270	DNA replication initiation	−6.40
SGDH284	GO:0051707	response to other organism	−7.89
	GO:1901605	alpha-amino acid metabolic process	−7.77
	GO:0044283	small molecule biosynthetic process	−7.66
	GO:0046394	carboxylic acid biosynthetic process	−7.52
	GO:0009605	response to external stimulus	−7.43
	GO:0009607	response to biotic stimulus	−6.55
	GO:0016998	cell wall macromolecule catabolic process	−6.31
	GO:0046348	amino sugar catabolic process	−5.92
	GO:0044710	single-organism metabolic process	−5.62
	GO:0006790	sulfur compound metabolic process	−5.42
top10 enriched GO terms for line-specific up-regulated genes			
158A	GO:0051707	response to other organism	−9.28
	GO:0051704	multi-organism process	−8.19
	GO:0009605	response to external stimulus	−8.18
	GO:0009607	response to biotic stimulus	−7.38
	GO:1901565	organonitrogen compound catabolic process	−6.68
	GO:1902578	single-organism localization	−5.62
	GO:0006855	drug transmembrane transport	−5.51
	GO:0042493	response to drug	−5.42
	GO:0055085	transmembrane transport	−5.07
	GO:0044765	single-organism transport	−4.74
SGDH284	GO:0042254	ribosome biogenesis	−9.72
	GO:0006396	RNA processing	−7.68
	GO:0034470	ncRNA processing	−6.64
	GO:0051503	adenine nucleotide transport	−4.82
	GO:0030163	protein catabolic process	−4.59
	GO:0044085	cellular component biogenesis	−4.54
	GO:0007275	multicellular organism development	−4.29
	GO:0009793	embryo development ending in seed dormancy	−4.27
	GO:0033523	histone H2B ubiquitination	−4.09
	GO:0016574	histone ubiquitination	−4.09

Some previously characterized COR genes in *B. napus* were found in the collected DEGs in this work. *BN28* (BnaAnng37980D, *Kin1*), *BN115* (BnaA03g56750D, *COR15*) and *BN59* (BnaA07g34490D, *VHA-A*) were the first characterized COR genes in *B. napus*, with their transcripts accumulated in cold stressed plants [6, 8, 16]. In this work, up-regulation of these genes were observed in both 158A and SGDH284, with the log2foldchanges ranged from 1.1 to 5.6. Earlier studies also revealed transcripts accumulation of four CBF-like genes in *B. napus* response to low temperature, e.g., *CBF5*, *CBF7*, *CBF16* and *CBF17* [10, 11]. *CBF5* (BnaC03g71900D), *CBF7* (BnaAnng34260D) and *CBF16* (BnaC07g39680D) were also up-regulated during low-temperature exposure of 158A and SGDH284 (log2foldchange ranged from 2.4–4.4). However, the exact locus for *CBF17* was not determined in the selected genome version (PRJEB5043_v1), which might be ascribed to the difference in gene model construction during genome annotation. The most similar gene to *CBF17* was BnaA08g30910D (80% identity), but it was not varied in expression in any cold stressed lines in this work.

In contrast, transcripts accumulation for some other known COR genes might not be essential for cold acclimation of 158A or SGDH284 in short-day condition. For example, previous reports revealed that *BnCOR25* and *hsp90* were induced in *B. napus* by cold stress [7, 9]. However, transcriptomics analysis in this work found that the expression of *BnCOR25* or *hsp90* was not significantly altered in either 158A or SGDH284 after low temperature exposure.

### Expression of TFs in cold stressed *B. napus*

TFs play crucial roles in plant response to various environmental stresses, e.g., in the well characterized model organism *A. thaliana*, more than a thousand stress responsive TFs were identified based on the integrated curation and genomic data mining approach [17]. In *B. napus*, a total of 5985 TFs were annotated, with many of them involved in stresses response, such as the CBFs and a series of NACs [10, 18]. However, a systematic identification of cold stress responsive TFs is still lack.

**Fig. 2:**
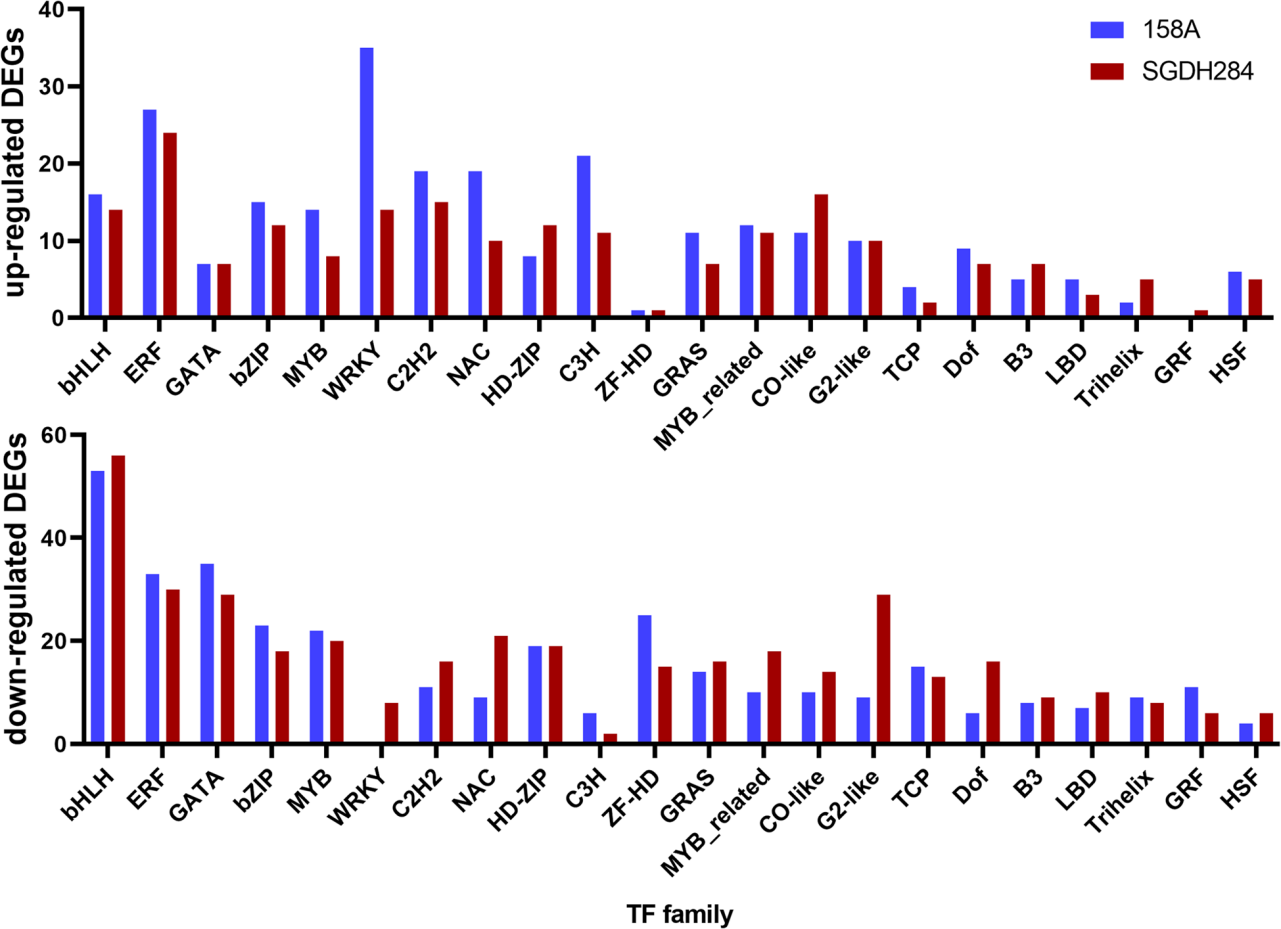
Statistics of differentially expressed transcription factors in 158A and SGDH284. Classification of transcription factors in *Brassica napus* was retrieved from PlantTFDB

In this work, derived from the estimated expression profiles of two *B. napus* lines, we found that expression of about 40% of the TFs were detectable (FPKM ≥1) in at least one of the investigated samples. 692 and 708 TFs were differentially expressed during low-temperature exposure of 158A and SGDH284, respectively, including 402 TFs shared by two lines. All the differentially expressed TFs belonged to 54 families (Additional file 1: Table S3 and Fig. 2). Of them, bHLH was the largest family that about 70 differentially expressed bHLH type TFs were identified in each line. However, it was CO-like family that had the highest ratio (about 50%) of varied expressed members. Interestingly, in the collected differentially expressed TFs, members of some families were mostly down-regulated (e.g., bHLH, GATA and ZF-HD) or up-regulated (e.g., WRKY, C3H and DBB) in both lines. Specifically, all of the differentially expressed WRKY (35 genes) were up-regulated in 158A, in contrast to that both up-regulated (14 genes) and down-regulated (8 genes) WRKY genes were found in SGDH284. In addition, in both 158A and SGDH284, almost all the differentially expressed GRFs (growth-regulation factors) were down-regulated.

### Identification of key regulators involved in cold response

Two strategies were used to identify the key regulators involved in *B. napus* response to cold stress. First, homology search identified 128 TFs that showed high sequence similarity (> 50% identity in amino acid) to the cold-responsive TFs in *A. thaliana* and were differentially expressed in 158A or/and SGDH284 after plants exposure to low temperature. 19 and 37 of them were down- (e.g., multiple ethylene-responsive transcription factors) and up-regulated (e.g., homologs of CBF1/2 and ZAT6/10/12) in both 158A and SGDH284, respectively (Table 2). Derived from the description in STIFDB (Stress Responsive Transcription Factor Database) [17], most of these 56 TFs were also involved in response to some other stresses, such as drought, light and salt.

**Table 2:** Homology based identification of cold responsive transcription factors in *Brassica napus*

Gene in *B. napus*	Homolog in A. thaliana^*^	Symbol	Identity%	log2foldchange^#^		Description	Stress elements^**^
				158A	SGDH284		
BnaCnng41320D	AT3G19290	ABF4	66.8	1.7	1.6	Abscisic acid responsive elements-binding factor 4	drought/NaCl/ABA/cold
BnaA05g11360D	AT2G31380	BBX25	88.7	2.7	1.6	B-box zinc finger protein 25	cold/light
BnaCnng49280D	AT4G25490	CBF1	65.6	5.2	7.2	Dehydration-responsive element-binding protein 1B	cold/drought/salt
BnaAnng34260D BnaC07g39680D BnaC03g71900D BnaA03g13620D	AT4G25470	CBF2	78.2 74.2 76.5 77.0	2.4 3.2 4.3 2.4	2.7 3.4 4.4 6.9	Dehydration-responsive element-binding protein 1C	cold/drought/salt
BnaC04g31720D	AT5G39660	CDF2	74.6	2.6	1.1	Cyclic dof factor 2	cold/drought/UV-B
BnaA02g02840D BnaC09g41980D BnaA10g18420D	AT5G15850	COL1	63.1 66.6 66.4	2.2 2.7 2.2	1.3 1.5 1.7	Zinc finger protein CONSTANS-LIKE 1	cold/ABA/drought/light/NaCl
BnaC03g35440D	AT3G07650	COL9	80.1 85.9	4.2 1.6	3.1 4.5	Zinc finger protein CONSTANS-LIKE 9	cold/NaCl
BnaA05g29980D							
BnaC03g22460D	AT2G40140	CZF1	75.6	5.0	2.2	Zinc finger CCCH domain -containing protein	cold
BnaC09g49920D	AT5G05410	DREB2A	64.7	1.8	2.9	Dehydration-responsive element-binding protein 2A	cold/drought/salt
BnaA10g25000D			62.2	2.8	3.8		
BnaA05g05080D	AT3G61150	HDG1	65.0	1.1	1.2	Homeobox-leucine zipper protein HDG1	cold
BnaCnng44540D	AT3G24310	MYB305	81.8	7.1	5.2	MYB-like DNA-binding domain protein	cold/NaCl
BnaA03g53110D	AT4G34990	MYB32	75.4	1.4	2.3	Transcription factor MYB32	cold/NaCl
BnaA01g16400D	AT4G27410	NAC072	87.0	2.3	1.4	NAC domain-containing protein 72	cold/drought/NaCl/ABA/light
BnaC08g04820D	AT1G46768	RAP2–1	78.1	3.4	2.6	Ethylene-responsive transcription factor RAP2–1	cold
BnaA05g25200D	AT3G14230	RAP2–2	73.6	1.8	1.3	Ethylene-responsive transcription factor RAP2–2	cold
BnaC05g39400D			74.1	1.8	1.6		
BnaA03g33290D			69.5	1.0	1.9		
BnaC03g08310D	AT5G17300	RVE1	78.0	2.7	1.9	Protein REVEILLE 1	cold/drought/salt
BnaA01g19870D	AT3G46600	SCL30	80.4	1.6	1.5	Scarecrow-like protein 30	cold/drought/UV-B/iron
BnaC01g24660D			70.6	1.3	1.8		
BnaA05g11640D	AT2G31070	TCP10	76.8	1.9	2.0	Transcription factor TCP10	cold
BnaC07g43320D	AT4G31550	WRKY11	72.0	1.5	1.1	Probable WRKY transcription factor 11	ABA/cold/light
BnaA04g22040D	AT2G38470	WRKY33	75.9	4.0	1.8	Probable WRKY transcription factor 33	cold/drought
BnaC03g58080D	AT1G27730	ZAT10	87.6	1.7	2.2	Zinc finger protein ZAT10	cold/drought/ABA/NaCl
BnaC07g16940D			61.8	7.1	3.6		
BnaA03g09250D	AT5G59820	ZAT12	85.2	1.4	1.1	Zinc finger protein ZAT12	cold/drought
BnaC09g35160D			84.7	2.6	2.4		
BnaA10g12780D			86.4	3.4	3.7		
BnaA10g25850D	AT5G04340	ZAT6	76.0	2.3	3.7	Zinc finger protein ZAT6	cold
BnaCnng20570D			72.0	4.0	7.7		
BnaA03g54460D	AT4G39780	ERF060	76.2	−2.7	−3.4	Ethylene-responsive transcription factor ERF060	cold
BnaC07g46940D			76.4	−2.3	−2.7		
BnaA09g55520D	AT3G61890	ATHB-12	78.9	−2.1	−2.0	Homeobox-leucine zipper protein ATHB-12	cold/drought/NaCl
BnaA05g24390D	AT3G15210	ERF4	72.7	−1.2	−2.2	Ethylene-responsive transcription factor 4	cold/drought
BnaA06g01090D			64.7	−2.5	− 2.0		
BnaC03g39000D			77.3	−1.4	−1.9		
BnaA03g33790D			76.4	−1.2	−1.8		
BnaC05g37390D	AT4G14540	HAP3C	85.1	−2.0	−2.6	Transcriptional activator HAP3C	cold
BnaC03g39380D			85.3	−2.3	− 2.3		
BnaA03g33970D			78.3	−1.9	−2.1		
BnaA01g28350D			85.5	−1.2	− 1.6		
BnaC02g25060D	AT1G78600	LZF1	83.7	−2.2	−2.1	B-box zinc finger protein 22	cold
BnaC08g48630D	AT3G50060	MYB77	72.1	−1.4	−1.0	Transcription factor MYB77	cold
BnaA07g13990D	AT2G28550	RAP2–7	80.9	−1.7	− 1.9	Ethylene-responsive transcription factor RAP2–7	cold/ABA/light
BnaC04g15640D			80.9	−1.4	−1.4		
BnaA06g07470D	AT1G13260	RAV1	85.5	−2.4	−2.5	Ethylene-responsive transcription factor RAV1	cold/NaCl
BnaAnng40580D BnaCnng37790D			84.6	−3.0	−2.4		
			84.9	−1.4	−1.7		
BnaA06g24950D	AT5G67580	TRB2	79.4	−1.1	−1.3	Telomere repeat-binding factor 2	cold/NaCl/drought

^*^Homology search was performed by comparing the predicted proteomes of *B. napus* and *A. thaliana*

^#^Log2foldchange indicates the expression variation between the cold stressed plants and the control. Genes were collected only when they were differentially expressed in both 158A and SGDH284 in the same manner

**Stress elements were assigned based on the description of *A. thaliana* genes in STIFDB

In addition, enrichment analysis was carried out to find the TFs with their targets over-represented in the total DEGs, resulting in 51 and 55 enriched TFs in line 158A and SGDH284, respectively (Additional file 1: Table S4). Twenty-five of these TFs were shared by two lines (Table 3), including several genes homologous to COR genes in other plants, such as CBF7, CAMTA1 and PIF4/7 that were involved in the CBF pathway, RVE7 and TCP21 in the regulation of the circadian clock by interacting with the CBF pathway components LHY and CCA1 [19, 20], BZIP44 and CDF5 in seed germination and flowering [21, 22], as well as some other genes associated with multiple stresses response. Notably, 1495 and 1520 potential targets of these 25 TFs were differentially expressed in 158A and SGDH284, accounting for about 15% of the total DEGs, respectively. Function analysis revealed that 16 out of these 25 TFs were enriched in biological processes like photosynthesis, plant development (e.g., seed germination, root development and circadian rhythm), amino acid catabolism and stimulus response (Table 3).

**Table 3:** Regulation based identification of key transcription factors involved in cold response of *Brassica napus*

Transcription factor^*^	Description^#^	Predicted function
**BnaC07g43590D**	two-component response regulator ARR10	NULL
**BnaC07g07840D**	transcription factor bHLH77	GO:0015979 photosynthesis GO:0019684 photosynthesis, light reaction GO:0009186 deoxyribonucleoside diphosphate metabolic process
**BnaC01g10420D**	transcription factor MYC4	NULL
**BnaAnng03730D**	ethylene-responsive transcription factor ERF011	GO:1901606 alpha-amino acid catabolic process GO:0006544 glycine metabolic process GO:1901565 organonitrogen compound catabolic process
**BnaA05g04450D**	ethylene-responsive transcription factor ERF034	NULL
**BnaA03g19970D**	transcription factor PIF4-like	GO:0015979 photosynthesis GO:0009853 photorespiration GO:0015977 carbon fixation
**BnaA09g37540D**	transcription factor PIF5-like	GO:0009853 photorespiration GO:0015977 carbon fixation GO:0015979 photosynthesis
**BnaA03g40080D**	transcription factor PIF7	GO:0015979 photosynthesis GO:0006091 generation of precursor metabolites and energy GO:0019684 photosynthesis, light reaction
**BnaA06g27900D**	ethylene-responsive transcription factor TINY	NULL
**BnaCnng16520D**	transcription factor DIVARICATA	NULL
**BnaC05g14070D**	REVEILLE 7	GO:0000160 phosphorelay signal transduction system GO:0050896 response to stimulus GO:0007623 circadian rhythm
**BnaA06g12480D**	REVEILLE 7	GO:0043086 negative regulation of catalytic activity GO:0010035 response to inorganic substance GO:0009892 negative regulation of metabolic process
**BnaA06g24950D**	telomere repeat-binding factor 2	GO:0009845 seed germination GO:0071103 DNA conformation change GO:0006091 generation of precursor metabolites and energy
**BnaC09g47560D**	transcription factor TCP21	GO:0033014 tetrapyrrole biosynthetic process GO:0046148 pigment biosynthetic process GO:0033013 tetrapyrrole metabolic process
**BnaA06g26010D**	transcription factor TCP7-like	GO:0015995 chlorophyll biosynthetic process GO:0018130 heterocycle biosynthetic process GO:0019438 aromatic compound biosynthetic process
BnaC06g22430D	bZIP transcription factor 44	GO:0015979 photosynthesis GO:0018298 protein-chromophore linkage GO:0009773 photosynthetic electron transport in photosystem I
BnaC08g05600D	bZIP transcription factor 60	NULL
BnaC05g17700D	transcription factor TGA3	NULL
BnaA01g37250D	protein indeterminate-domain 11	GO:0016114 terpenoid biosynthetic process GO:0006720 isoprenoid metabolic process GO:0008610 lipid biosynthetic process
BnaA10g22560D	calmodulin-binding transcription activator 1	GO:0080022 primary root development GO:0016052 carbohydrate catabolic process GO:0009664 plant-type cell wall organization
BnaA07g24230D	cyclic dof factor 5	GO:0006355 regulation of transcription, DNA-templated GO:0008643 carbohydrate transport
BnaAnng34260D	CBF-7	GO:0016998 cell wall macromolecule catabolic process GO:0046348 amino sugar catabolic process GO:0006040 amino sugar metabolic process
BnaC08g04820D	ethylene-responsive transcription factor RAP2–1	NULL
BnaC07g29370D	ethylene-responsive transcription factor SHINE 3	GO:0015979 photosynthesis GO:0018298 protein-chromophore linkage GO:0009817 defense response to fungus, incompatible interaction
BnaC07g13550D	NAC domain-containing protein 13	NULL

^*^Transcription factors were collected if their potential targets were over-represented in the total differentially expressed genes in both 158A and SGDH284

^#^Possible function of these genes in cold stressed *B. napus* were predicted by analysis of their differentially expressed targets (top three GO terms were shown). Bold identifiers indicated the down-regulated genes, while the others were up-regulated genes in cold stressed plants

**Fig. 3:**
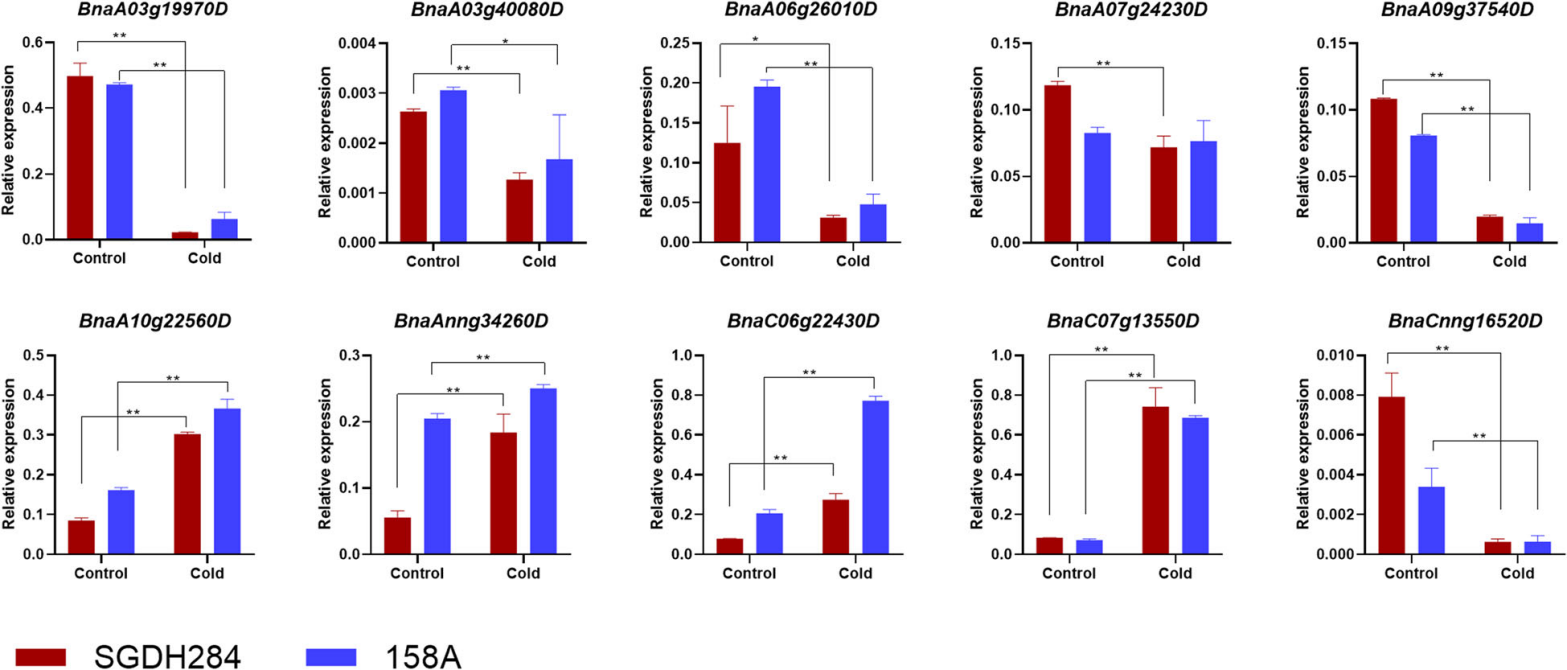
Differential expression of ten transcription factors in response of *Brassica napus* to cold stress. * (*p* < 0.05) and ** (*p* < 0.01) indicated the significance of differential expression

Summarily, homology search and TF enrichment based strategies identified 56 and 25 TFs, respectively, which were up−/down-regulated in both 158A and SGDH284. Ten of them were selected and their expression variation under cold stress condition was validated by RT-qPCR (Fig. 3 and Additional file 1: Table S5).

**Fig. 4:**
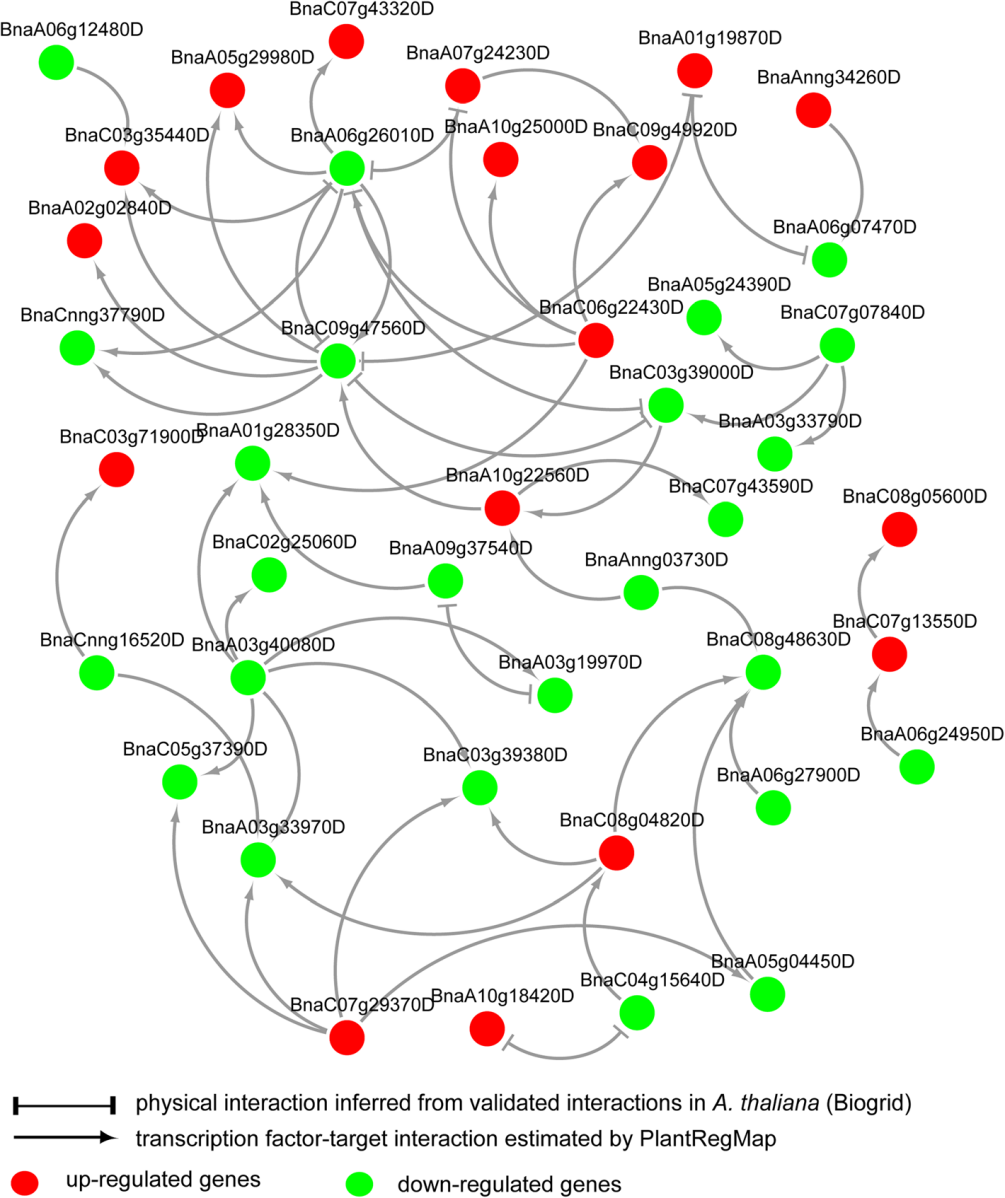
Interaction networks of identified cold responsive transcription factors in *Brassica napus*. Transcription factors were identified by using homology search and TF enrichment analysis. Interactions were estimated based on the protein-protein interactions in *Arabidopsis thaliana* and the reciprocal best hit pairs between *A. thaliana* and *B. napus*, as well as the regulations predicted in *B. napus*. All these genes were regulated in the same manner in 158A and SGDH284

Moreover, inferred from the curated protein-protein actions in *A. thaliana* and the predicted regulation in *B. napus*, a regulatory network of the identified cold-responsive TFs was built (Fig. 4). According to the constructed networks, some TFs worked at the initial steps of regulation, such as BnaAnng34260D (CBF7) and BnaC07g07840D (BHLH77), while some TFs regulated the expression of multiple other TFs, e.g., BnaA06g26010D (TCP7) and BnaC09g47560D (TCP21) and BnaA03g40080D (PIF7).

## Discussion

In an attempt to further understanding the molecular mechanisms of *B. napus* response to cold stress, herein we analyzed the gene expression change in cold stressed semi-winter type and winter type *B. napus* lines. Similar to the findings in other plants that about 10–15% genes were differentially expressed in response to cold stress [23–25], global modulation of gene expression was also observed in *B. napus* lines 158A and SGDH284 after low temperature exposure. Unexpectedly, despite of the high similarity in genetics between 158A and SGDH284, only 60% of the DEGs were shared. The shared DEGs indicated some common biochemical and physiological features of cold stressed plants, such as enhanced ability of stresses tolerance and inhibited photosynthesis. Cold acclimation accompanied inhibition of photosynthesis was observed in many plants, due to the effects of cold on electronic transport and carbon fixation, as well as the availability of free phosphate in the chloroplast, which could be assessed by the content of chlorophyll a [26]. In addition, further inspection of the about 40% line-specific DEGs demonstrated significantly varied gene regulatory mechanisms in cold stressed 158A and SGDH284. Stresses responsive related genes were enriched in both sets of line-specific DEGs, but were oppositely regulated in 158A and SGDH284. Similarly, biological processes related to the basic cellular activities were significantly inhibited in 158A but enhanced in SGDH284. This was consistent with the fact that growth of semi-winter type *B. napus* plants were more significantly inhibited than the winter cultivars once encountered extremely cold temperature in the field. Interestingly, histone H2B ubiquitination related genes were enriched in SGDH284 specific genes. Previous reports revealed that H2B monoubiquitination was related to the activation of transcription, which was related to the regulation of cuticle composition that could protect the plants from abiotic/biotic stresses, and also contributed to the plant growth fitness by modulating the expression of circadian clock genes [27–29]. It seems histone H2B ubiquitination played crucial roles in SGDH284 adaptation to low temperature survival based on this work, however, further investigations are needed for exploration of the exact roles and mechanisms.

Expression of previously characterized COR genes were checked in this work. Most of them were also up-regulated in both semi-winter and winter type *B. napus*, which was consistent with the previous reports. But some of them, like *BnCOR25* and *hsp90*, were not altered in expression in any line. We have noticed that, in contrast to a short-day photoperiod in this work, accumulation of *BnCOR25* and *hsp90* in cold stressed *B. napus* was detected under the 16 h light/8 h dark cycle [7, 9]. Thus further investigation is required to explore whether these differences is caused by the photoperiod.

Derived from the comparative genomics analysis in this work, members of particular TF families tended to be up- or down-regulated in cold acclimation of *B. napus*, such as WRKY and GRF genes, respectively. WRKY genes were involved in plant response to kinds of environmental stresses [30, 31], and up-regulation of some WRKY genes in *B. napus* response to multiple stresses was previously observed [32, 33]. In the semi-winter type line 158A, all the differentially expressed WRKY genes were up-regulated, indicating their crucial roles in cold tolerance of 158A. Moreover, GRFs usually played a role in the regulation of cell expansion in leaf and cotyledon [34], and in a semi-winter type *B. napus* zy036, the role of GRF2 in enhancing seed oil production was observed, which possibly worked by regulating cell number and plant photosynthesis [35]. Herein, down-regulation of GRF2a and GRF2b (BnaA01g00300D/BnaC07g46760D) was observed in both semi-winter and winter type lines, indicating their conserved roles in regulation of photosynthesis. More down-regulated GRF genes were found in 158A, which coincided with the phenotype that winter type *B. napus* usually exhibit stronger physiological activity after cold stress and that the growth of semi-winter type *B. napus* lines were more severely restricted by cold condition. However, whether these facts are directly associated with the different cold responsive mechanisms between semi-winter and winter type rapeseeds remains to be cleared.

56 and 25 cold responsive TFs were subsequently predicted based on homology search and enrichment analysis, respectively, which were regulated in the same manner in both semi-winter and winter type *B. napus* lines. Inferred from the functional analysis of target genes and the reconstructed transcriptional cascades, several TFs were assumed to be of great importance in modulating the gene expression to enhance the cold tolerance of *B. napus*, e.g., CBF7, BHLH77, TCP7, TCP21 and PIF7, including some well characterized COR genes like CBF7 and PIF7, indicating our strategy was an effective method for exploration of key regulators.

## Conclusions

Extensive biochemical and physiological adjustment and gene expression modulation are required for plants in response to low temperature. Some genes and pathways play similar roles across plant species, such as CBFs and photosynthesis/chloroplast related genes [36]. However, widespread variation in gene expression in cold stressed plants was also observed, which was largely ascribed to the inherent genetic difference. In order to identify the conserved genes and pathways involved in *B. napus* response to cold stress, we compared the transcriptional changes in semi-winter type line 158A and winter type line SGDH284. Enhanced stresses response mechanisms and inhibited photosynthesis were observed in both lines, indicating the common features of *B. napus* cold acclimation were conserved within species. Nevertheless, we also found that about 40% DEGs were line specific, suggesting the remodeling of regulatory mechanisms occurred during environmental adaptation. Moreover, involvement about eighty key TFs in *B. napus* cold response were predicted by an integrated strategy. These regulators covered main characterized cold response mechanisms and were responsible for expression variation of a great number of genes, thus provided a valuable collection for comprehensive understanding of the stress responsive mechanisms in *B. napus*.

Apart from transcriptomics analysis performed in this work and elsewhere, the other strategies like genome-wide association study (GWAS) have also played crucial roles in identification of genes related to agricultural traits [37–39], as well as the locus/genes involved in plants response to varied environmental stresses like low temperature [40–42]. Considering the characteristics of transcriptomes based analysis, e.g., they are usually used to assess tissue-specific and stage-specific regulations, application of multi-dimensional research methods including transcriptomics, GWAS and the integrated strategy such as expression quantitative trait loci (eQTL) are therefore expected for systematically depiction of the cold stress mechanisms in *B. napus* in future.

## Methods

### Plant materials and growth conditions

*Brassica napus* SGDH284 and 158A, two DH lines selected in our lab from microspore culture of winter rapeseed *Sollux* and semi-winter rapeseed *zhongyou9988*, respectively, were used in this study. *Sollux* was kindly provided by Dr. Xiyuan Ni from Zhejiang Academy of Agricultural Sciences, and *zhongyou9988* is a commercial variety in China selected by Oil Institute of Chinese Academy of Agricultural Sciences. Seeds were planted in 4 × 8 hole flowerpots (54 × 28 × 7 cm) containing soil substrates and pearlite with a ratio of 3:1. The flowerpots were transferred into a plant growth chamber (Percival E-36 L) under a short-day photoperiod (10-h light/14-h dark) and a humidity of 60% at 25 °C and were grown for 4 weeks since germination. For cold treatment two trays of the seedlings of each lines were transferred to another plant growth chamber at 4 °C under the same photoperiod and humidity for 7 days. Another four trays of seedlings were set as the control group and were left at the same plant growth chamber with the same light and temperature condition. After treatments, the fully expanded first leaves of 6–8 plants from the same trays were collected and pooled as one sample. The leaves were frozen in liquid nitrogen as soon as they were cut down from the plants. Then the samples were stored at − 80 °C for further analysis.

### RNA extraction and RNA-seq

Total RNA was isolated using the TRIzol regent (Invitrogen, Carlsbad, USA) according to the manufacturer’s instructions. The RNA quantity and quality were checked by gel electrophoresis and a DU800 spectrophotometer (BECKMAN, USA). A total of 50 μg RNA (1000 ng/μL) for each sample was used for RNA sequencing. The Illumina NextSeq 500 platform was applied for RNA-seq. Eight libraries were constructed and paired-end sequencing was performed according to the manufacturer’s instructions (TruSeq RNA Sample Prep Kit, Illumina). Shortly, magnetic beads with oligo poly (T) attached were used for purifying the mRNA, which were cut into short fragments of about 375 bp in fragmentation buffer. Then the purified fragmented mRNA were converted into double-stranded cDNA and adapters were added to both end of the short fragments. AMPure XP beads were used to remove the unsuitable fragments and by PCR amplification the sequencing libraries were constructed. The libraries were normalized and loaded to the Illumina NextSeq 500 platform (Shanghai Personal Biotechnology Co Ltd, Shanghai, China) for sequencing after being checked with Pico green staining (PicoGreen dsDNA assay kit, Invitrogen, P7589) and fluorospectrophotometry (Quantiflour-ST fluorometer, Promega, E6090), and quantified with Agilent 2100 (Agilent 2100 Bioanalyzer, Agilent 2100; Agilent High Sensitivity DNA Kit, Agilent, 5067–4626).

### Identification of differentially expressed genes

Quality control of raw reads generated from Illumina platform were performed by using Trimmomatic (version 0.33) (LEADING:20, TRAILING:20 SLIDINGWINDOW:4:20, MINLEN:25) [43]. Clean reads were then mapped to the reference genome of *Brassica napus* from EnsemblPlants (http://plants.ensembl.org, release 42) with TopHat (v2.1.0) (default setting) [44]. Cufflinks (v2.2.1) was used to reconstruct the transcriptomes and calculated the expression level. Only genes with their FPKM (Fragments Per Kilo bases per Million reads) ≥ 1 in at least one samples were collected for further analysis. HTseq-count (version 0.11.2) was used to calculated the number of short reads mapped to the characterized gene locus and DEseq2 was used for identification of differentially expressed genes (DEGs) (foldchange ≥2 and *p*-value ≤0.05) [45, 46].

### Functional enrichment analysis

GO enrichment analysis in this work was carried out using the tools provided by PlantRegMap (http://plantregmap.cbi.pku.edu.cn) [47]. The online server REViGO (http://revigo.irb.hr/) was used to remove the redundancy of GO terms [48].

### Identification of cold responsive transcription factors

Homology based identification of cold responsive TFs was performed by comparing the *B. napus* genes to the stresses responsive TFs in *A. thaliana* with the BLASTP tool (cutoff identity 50% and coverage 50% for both query and hit sequences in length). Stresses responsive TFs in *A. thaliana* were retrieved and downloaded from the STIFDB (http://caps.ncbs.res.in/stifdb2/) [17]. In addition, TF enrichment analysis was performed through PlantRegMap with the tool ‘TF Enrichment’ (cutoff p-value 0.05) [47]. Briefly, transcriptional regulations in *B. napus* were firstly identified based on the genome-wide analysis of functional transcription factor binding sites (FunTFBS). Subsequently, Fisher’s exactly test was performed to identify the TFs with their targets over-represented in the total DEGs in the cold stressed 158A and SGDH284, respectively.

### Interaction networks of cold responsive TFs

Firstly, interactions between *A. thaliana* proteins were retrieved from the BioGRID [49]. Protein-protein interactions for TFs in *B. napus* were then predicted according to the estimated BLAST reciprocal best hits between *A. thaliana* and *B. napus* in PlantRegMap. Potential regulations between the TFs in *B. napus* were retrieved from PlantRegMap (FunTFBS method), which were then merged with the predicted protein-protein interactions using in house developed perl scripts. Cytoscape (3.4.0) was used to display and analyze the constructed networks [50].

### Quantitative real-time PCR analysis

To validate the expression profiles of genes, ten randomly selected DEGs were used to perform Quantitative Real-Time PCR (qRT-PCR) analysis. Three samples of each treatment were harvested for total RNAs extraction, which were different from those used in RNA-seq. After reverse-transcribed with FastQuant RT Kit (TIANGEN Biotech, Beijing, China), the qRT-PCR reactions were performed with THUNDERBIRD SYBR qPCR Mix (TOYOBO, Osaka, Japan) and a Mastercycler ep realplex (Eppendorf, Germany) according to the manufacturer’s instructions. The thermal cycling conditions were as follows: 94 °C for 2 min, followed by 40 cycles of 10 s at 94 °C and 20 s at 60 °C, then 72 °C for 20 s. All reactions were performed in triplicate, and the *actin7* was used as an internal reference gene (accession number: Bra028615) [51]. The relative gene expression levels were calculated by using the 2 − ΔΔCt method [52].

### Statistics

Elsewise specified, *chi*-square test was used in this work to calculate the significance of gene number variation between different groups.

## Supplementary information

**Additional file 1: Table S1.** Statistics of RNA-seq and short reads mapping. **Table S2.** Significantly varied GO terms between 158A and SGDH284 for line specific differentially expressed genes. **Table S3.** Statistics of differentially expressed transcription factors in 158A and SGDH284. **Table S4.** Enrichment analysis of cold responsive transcription factors in 158A and SGDH284. **Table S5.** Primer pairs used to detect the expression of selected transcription factors.

## Data Availability

Raw data generated by this work is available in BIGD under the project PRJCA001621 (https://bigd.big.ac.cn/bioproject/browse/PRJCA001621).

## Abbreviations

CBFs: Cold-binding transcription factors
COR: Cold-responsive
DEGs: Differentially expressed genes
DREBs: Dehydration responsive element binding factors
GRFs: Growth-regulation factors.
TFs: Transcription factors
