# Supplementary material for: Genome-wide identification of cold responsive transcription factors in Brassica napus L
Source: BMC Plant Biol. 2020 Feb 6;20:62. doi: 10.1186/s12870-020-2253-5 (PMC7006134; doi:10.1186/s12870-020-2253-5)
Supplement: Supplementary file 1 — Additional file 1: Table S1. Statistics of RNA-seq and short reads mapping. Table S2. Significantly varied GO terms between 158A and SGDH284 for line specific differentially expressed genes. Table S3. Statistics of differentially expressed transcription factors in 158A and SGDH284. Table S4. Enrichment analysis of cold responsive transcription factors in 158A and SGDH284. Table S5. Primer pairs used to detect the expression of selected transcription factors. [file 12870_2020_2253_MOESM1_ESM.pdf]

Table S1. Statistics of RNA-seq and short reads mapping.

Dataset	Raw reads	Clean reads	Mapped reads	Mapped ratio	mapped pairs
**158A-1(control-1)**	76527310	66650730	46568448	69.90%	19903089
**158A-2(control-2)**	83663158	73300196	50786536	69.30%	21596005
**158A-3 (treated-1)**	55300492	48032344	33995080	70.80%	14579943
**158A-4(treated-2)**	56028358	49207616	36624084	74.40%	16384087
**SGDH284-1(control-1)**	93993476	79504702	57191752	71.90%	24748565
**SGDH284-2(control-2)**	67991778	59220656	42652638	72.00%	18411462
**SGDH284-3 (treated-1)**	83014900	71982590	50543747	70.20%	21602395
**SGDH284-4 (treated-2)**	83766942	72461866	51002997	70.40%	21872507

Table S2. Significantly varied GO terms between 158A and SGDH284 for line specific differentially expressed genes.

Class	GO terms	Number of genes in line		p-value
		158A	SGDH284	
down-regulated	translation	138	71	1.67E-06
	microtubule-based movement	32	12	1.92E-03
	DNA replication	20	6	6.82E-03
	regulation of cell cycle	15	4	1.27E-02
	regulation of cyclin-dependent protein serine/threonine kinase activity	17	6	2.66E-02
	leucine biosynthetic process	5	0	4.78E-02
up-regulated	drug transmembrane transport	27	7	5.27E-04
	transmembrane transport	162	122	1.54E-02
	protein phosphorylation	189	151	3.95E-02
	cell redox homeostasis	33	18	4.21E-02
	ribosome biogenesis	6	16	3.70E-02

Table S3. Statistics of differentially expressed transcription factors in 158A and SGDH284.

Family	Gene numbers in 158A			Gene numbers in SGDH284			Total_in_geno
	up-regulated	down-regulated	total_in_DEG	up-regulated	down-regulated	total_in_DEG	
**bHLH**	16	53	69	14	56	70	553
**ERF**	27	33	60	24	30	54	449
**GATA**	7	35	42	7	29	36	125
**bZIP**	15	23	38	12	18	30	264
**MYB**	14	22	36	8	20	28	489
**WRKY**	35	0	35	14	8	22	285
**C2H2**	19	11	30	15	16	31	368
**NAC**	19	9	28	10	21	31	411
**HD-ZIP**	8	19	27	12	19	31	178
**C3H**	21	6	27	11	2	13	156
**ZF-HD**	1	25	26	1	15	16	62
**GRAS**	11	14	25	7	16	23	105
**MYB_related**	12	10	22	11	18	29	251
**CO-like**	11	10	21	16	14	30	48
**G2-like**	10	9	19	10	29	39	169
**TCP**	4	15	19	2	13	15	76
**Dof**	9	6	15	7	16	23	156
**B3**	5	8	13	7	9	16	211
**LBD**	5	7	12	3	10	13	136
**Trihelix**	2	9	11	5	8	13	100
**GRF**	0	11	11	1	6	7	34
**HSF**	6	4	10	5	6	11	96
**DBB**	7	1	8	9	2	11	33
**MIKC_MAD**	3	5	8	4	4	8	155
**NF-YB**	3	5	8	3	9	12	48
**BES1**	0	7	7	1	7	8	30
**AP2**	2	4	6	4	4	8	57
**Nin-like**	3	3	6	2	3	5	55
**SBP**	1	4	5	3	7	10	63
**ARR-B**	2	3	5	2	3	5	44
**NF-YC**	1	4	5	2	4	6	27
**CAMTA**	4	0	4	7	0	7	24
**RAV**	0	4	4	3	3	6	27
**TALE**	4	0	4	2	6	8	70
**E2F/DP**	3	0	3	1	0	1	32
**HB-other**	2	0	2	3	0	3	19
**WOX**	0	2	2	2	0	2	58
**NF-YA**	1	1	2	1	1	2	40
**M-type_MAI**	0	2	2	1	2	3	151
**SRS**	0	2	2	0	0	0	36
**FAR1**	0	2	2	0	0	0	12
**LSD**	0	2	2	0	3	3	13
**ARF**	1	0	1	5	1	6	64
**Whirly**	1	0	1	3	0	3	9
**GeBP**	0	1	1	2	0	2	38

**S1Fa-like**	0	1	1	1	0	1	12
**STAT**	1	0	1	0	0	0	2
**EIL**	1	0	1	0	0	0	23
**HRT-like**	0	1	1	0	1	1	5
**CPP**	0	1	1	0	0	0	34
**BBR-BPC**	0	1	1	0	1	1	26
**YABBY**	0	0	0	2	0	2	22
**HB-PHD**	0	0	0	1	0	1	8
**SRS**	0	0	0	0	2	2	36

*DEG: differentially expressed genes.

Classification of transcription factors in *Brassica napus* was retrieved from PlantTFDB.

Table S4. Enriched transcription factors in 158A and SGDH284.

Table S4-1. Enriched transcription factors in 158A.

Transcription factor	Family	description	Variation	pvalue	
				up-regulated	down-regulated
BnaC06g00880D	ERF	ethylene-responsive transcription factor	down-regulated	NA	1.93E-02
BnaA10g22930D	MYB	transcription factor DIVARICATA		NA	1.78E-36
BnaA03g40080D	bHLH	transcription factor PIF7		8.03E-15	2.14E-41
BnaA03g19970D	bHLH	transcription factor PIF4-like		5.52E-04	6.14E-22
BnaA06g12480D	MYB_related	REVEILLE 7		7.01E-23	NA
BnaC02g39310D	bZIP	basic leucine zipper 63		7.11E-08	2.25E-10
BnaA09g37540D	bHLH	transcription factor PIF5-like		NA	1.54E-07
BnaC09g47560D	TCP	transcription factor TCP21		3.89E-04	3.36E-21
BnaA06g26010D	TCP	transcription factor TCP7-like		NA	8.34E-09
BnaA06g27900D	ERF	ethylene-responsive transcription factor TINY		NA	1.73E-03
BnaC07g43590D	ARR-B	two-component response regulator ARR10		NA	4.95E-02
BnaC05g14070D	MYB_related	REVEILLE 7		1.53E-16	NA
BnaCnng16520D	MYB	transcription factor DIVARICATA		NA	5.37E-20
BnaAnng03730D	ERF	ethylene-responsive transcription factor		NA	1.20E-03
BnaC05g07080D	bHLH	transcription factor PIF3		1.54E-03	1.31E-22
BnaC07g07840D	bHLH	transcription factor bHLH77		1.02E-09	4.18E-09
BnaA06g24950D	MYB_related	telomere repeat-binding factor 2		4.04E-06	NA
BnaA05g04450D	ERF	ethylene-responsive transcription factor ERF034		NA	1.11E-02
BnaA07g24590D	ERF	ethylene-responsive transcription factor ERF118-like		1.63E-02	NA
BnaC01g10420D	bHLH	transcription factor MYC4		NA	1.57E-02
BnaA04g14500D	WRKY	WRKY transcription factor 17		1.72E-02	NA
BnaC05g17700D	bZIP	transcription factor TGA3		1.06E-05	NA
BnaC08g06930D	C2H2	SENSITIVE TO PROTON RHIZOTOXICITY 1-like		NA	4.10E-02
BnaCnng08620D	ERF	ethylene-responsive transcription factor ERF023		NA	4.28E-02
BnaA05g01520D	bZIP	G-box-binding factor 3		2.55E-14	1.76E-36
BnaC08g05600D	bZIP	bZIP transcription factor 60		2.21E-02	8.47E-03
BnaCnng77210D	ERF	ethylene-responsive transcription factor ERF039		NA	3.75E-03
BnaA10g22560D	CAMTA	calmodulin-binding transcription activator 1		NA	3.68E-04
BnaA01g31650D	NAC	NAC domain-containing protein 53		5.25E-03	NA
BnaC05g00840D	MYB_related	LHY-like		2.61E-06	NA
BnaC06g22430D	bZIP	bZIP transcription factor 44		1.55E-11	1.56E-25

BnaA06g22640D	C3H	zinc finger CCCH domain-containing protein 67	up-regulated	NA	1.74E-02
BnaA01g37250D	C2H2	protein indeterminate-domain 11		4.32E-02	4.40E-02
BnaA04g13570D	WRKY	WRKY transcription factor 15		7.29E-05	NA
BnaC03g58080D	C2H2	zinc finger protein ZAT10		3.51E-02	NA
BnaC03g21360D	WRKY	WRKY transcription factor 33		5.01E-07	NA
BnaC07g29370D	ERF	ethylene-responsive transcription factor SHINE 3		NA	5.00E-03
BnaA02g03510D	MYB_related	REVEILLE 1		2.38E-28	NA
BnaC01g16120D	WRKY	WRKY transcription factor 7		9.09E-05	NA
BnaC04g38910D	WRKY	WRKY transcription factor 45		3.59E-06	NA
BnaC03g11590D	MYB	transcription factor MYB59		3.79E-03	NA
BnaAnng34260D	ERF	CBF-7		1.35E-10	NA
BnaA08g04090D	ERF	ethylene-responsive transcription factor RAP2-1		NA	5.61E-04
BnaC09g13680D	WRKY	WRKY transcription factor 6		2.50E-03	NA
BnaA07g24230D	Dof	cyclic dof factor 5		1.73E-02	2.00E-02
BnaC07g35130D	WRKY	WRKY transcription factor 28		1.08E-03	NA
BnaC08g04820D	ERF	ethylene-responsive transcription factor RAP2-1		NA	5.61E-04
BnaC07g13550D	NAC	NAC domain-containing protein 13		2.05E-02	NA
BnaC05g38150D	NAC	NAC domain-containing protein 55		2.95E-04	NA
BnaA06g17950D	G2-like	transcription factor LUX		7.45E-03	NA
BnaC01g13500D	WRKY	WRKY transcription factor 31		1.59E-04	NA

Transcription factors were selected if their targets were over-represented in the total differentially expressed genes in cold stressed *B. napus* plants.

Table S4-2. Enriched transcription factors in SGDH284.

Transcription factor	Family	Description	Variation	pvalue	
				up-regulated	down-regulated
BnaA03g19970D	bHLH	transcription factor PIF4-like	down-regulated	1.21E-03	4.83E-23
BnaA08g22580D	MYB	MYB transcription factor 51		1.78E-02	4.53E-07
BnaA03g40080D	bHLH	transcription factor PIF7		2.56E-14	6.75E-64
BnaC04g07080D	G2-like	myb family transcription factor PHL8-like		NA	2.16E-02
BnaA05g28870D	MYB_related	REVEILLE 8		5.50E-30	NA
BnaC07g29050D	ERF	ethylene-responsive transcription factor TINY		NA	3.74E-03
BnaA10g24770D	MYB	transcription factor DIVARICATA-like		NA	9.60E-13
BnaCnng16520D	MYB	transcription factor DIVARICATA		NA	7.78E-08
BnaC02g30930D	ERF	ethylene-responsive transcription factor 9		2.24E-02	NA
BnaA06g26010D	TCP	transcription factor TCP7-like		NA	1.21E-05
BnaA09g37540D	bHLH	transcription factor PIF5-like		NA	6.59E-10
BnaC09g47560D	TCP	transcription factor TCP21		4.34E-04	3.22E-17
BnaC05g14070D	MYB_related	REVEILLE 7		2.77E-28	NA
BnaA08g28740D	bZIP	transcription factor RF2b		4.69E-04	NA
BnaC03g39000D	ERF	ethylene-responsive transcription factor 4		4.68E-03	NA
BnaA02g35660D	G2-like	transcription factor HHO2-like		NA	2.88E-03
BnaA01g30340D	G2-like	myb family transcription factor PHL12-like		4.38E-02	NA
BnaC09g05850D	Dof	dof zinc finger protein DOF5.6-like		4.98E-03	NA
BnaC07g43590D	ARR-B	two-component response regulator ARR10		2.91E-02	1.22E-02
BnaAnng03730D	ERF	ethylene-responsive transcription factor		NA	7.15E-03
BnaA05g04450D	ERF	ethylene-responsive transcription factor ERF034		NA	2.20E-02
BnaA03g39760D	Dof	dof zinc finger protein DOF5.4		1.54E-02	NA
BnaC09g12040D	Dof	dof zinc finger protein DOF1.8		1.29E-02	NA
BnaA06g12480D	MYB_related	REVEILLE 7		5.72E-35	NA
BnaC03g65930D	bZIP	bZIP transcription factor 11		1.59E-06	2.05E-26
BnaAnng39140D	MYB_related	transcription factor DIVARICATA-like		NA	2.63E-15
BnaA04g24390D	bHLH	transcription factor bHLH130		4.01E-04	NA
BnaA06g24950D	MYB_related	telomere repeat-binding factor 2		1.07E-10	NA
BnaA07g18730D	GATA	GATA transcription factor 4-like		1.67E-02	NA
BnaA06g27900D	ERF	ethylene-responsive transcription factor TINY		NA	3.74E-03
BnaC07g07840D	bHLH	transcription factor bHLH77		3.07E-09	5.76E-09
BnaAnng00370D	Dof	dof zinc finger protein DOF5.1		NA	1.09E-03

BnaC01g10420D	bHLH	transcription factor MYC4		NA	3.49E-02
BnaCnng05590D	HD-ZIP	homeobox-leucine zipper protein HAT5-like		NA	3.11E-02
BnaA10g23230D	bHLH	transcription factor BIM1		2.08E-03	9.91E-05
BnaCnng15140D	Dof	cyclic dof factor 3		1.13E-02	4.64E-03
BnaC09g52680D	HSF	heat stress transcription factor B-2b		NA	2.43E-02
BnaA03g43770D	BES1	BES1/BZR1 homolog protein 3	up-regulated	1.06E-03	2.77E-05
BnaC05g17700D	bZIP	transcription factor TGA3		2.79E-03	1.45E-05
BnaC07g29370D	ERF	ethylene-responsive transcription factor SHINE 3		NA	1.06E-02
BnaC08g05600D	bZIP	bZIP transcription factor 60		2.25E-02	2.28E-04
BnaC07g07130D	HSF	heat stress transcription factor C-1-like		NA	1.77E-03
BnaA05g08020D	bZIP	protein ABSCISIC ACID-INSENSITIVE 5		9.89E-22	3.78E-64
BnaA05g27620D	MYB	transcription factor MYB65-like		4.66E-02	NA
BnaA02g26190D	C2H2	protein indeterminate-domain 5, chloroplastic-like		1.70E-03	NA
BnaA08g00990D	ERF	ethylene-responsive transcription factor RAP2-12		2.65E-04	NA
BnaA01g37250D	C2H2	protein indeterminate-domain 11		NA	4.74E-02
BnaA07g24230D	Dof	cyclic dof factor 5		8.62E-03	2.15E-02
BnaC06g22430D	bZIP	bZIP transcription factor 44		9.55E-08	3.82E-36
BnaC08g04820D	ERF	ethylene-responsive transcription factor RAP2-1		NA	8.70E-03
BnaAnng34260D	ERF	CBF-7		2.50E-08	NA
BnaC07g13550D	NAC	NAC domain-containing protein 13		2.47E-03	NA
BnaA10g22560D	CAMTA	calmodulin-binding transcription activator 1		NA	2.17E-07
BnaC03g48820D	AP2	AP2-like ethylene-responsive transcription factor AIL7		1.77E-02	NA
BnaC07g10480D	ERF	ethylene-responsive transcription factor 12-like		2.95E-02	NA

Transcription factors were selected if their targets were over-represented in the total differentially expressed genes in cold stressed *B. napus* plants.

Table S5. Primer pairs used to detect the expression of selected transcription factors.

Genes	Primer pairs	
	Forward	Reverse
BnaA03g19970D	GGCTCTACTCGAACGAAAATTC	TGGTTCGACTTGTTATTACCGA
BnaA03g40080D	AAACCATTGTTATTGCAGGGAC	TGAGCTCTTTCACTCCGTAATT
BnaA06g26010D	GAAAACGAACCCAACTGATTGA	GGTGTTTGTGAAACGTATGTGA
BnaA07g24230D	ACTATTTCCACTTCCACGAACT	GAATCCTCTCTCAGGCGTTTTC
BnaA09g37540D	TTACGTGTGAAACAGAAAGCAG	CCAGCAATTCAGGAGATAGTGA
BnaA10g22560D	GAAAACGATTCAAGACCAACCA	GAATCTGCTACTTTCTTGCTCG
BnaAnng34260D	CAATTCCTGTGTGTGTTTTCGA	GAGTAGATCACGTTGTTGGAGA
BnaC06g22430D	CGAGGAGTCCATGTTAGAGATG	AAAAGGCAGAATCCGAAAATCC
BnaC07g13550D	AGAACCAAGATAGTAACTGGCC	CTGACTTGACTCGTCTTTTGTG
BnaCnng16520D	CGAGAAAATCATTTTGCGTTGG	GAGTCTACGGTATTGATCACGT
Actin7	GCTGACCGTATGAGCAAAG	AAGATGGATGGACCCGAC
